# A dataset of fine-grained zebrafish interactions in health and disease

**DOI:** 10.1038/s41597-026-06953-6

**Published:** 2026-03-03

**Authors:** Kosmas Deligkaris, Radmila Neiman, Makoto Hiroi, Tatsuo Izawa, Liam O’Shaughnessy, Luis Carretero Rodriguez, Ichiro Masai, Greg J. Stephens

**Affiliations:** 1 Okinawa Institute of Science and Technology Graduate University, Okinawa, Japan; 2 Department of Physics and Astronomy, Vrije Universiteit Amsterdam, Amsterdam, The Netherlands

## Abstract

Zebrafish (*Danio rerio*) has emerged as a valuable vertebrate model organism for studies of social interactions. While previous multiple-animal research has focused on gross movement, here we present a machine vision workflow to capture and analyze fine-grained social interactions through the tracking of three anatomical landmarks (at the head, pectoral fins, and tail) as well as the identity of the fish in 3D. We release a dataset of *N* = 173 five-hour recordings of adult zebrafish dyads, including male/male and female/female wild-type pairs and disease-model mutants, sampling complex behaviors such as dominance contests and aggressive/submissive motifs. The recordings are of high temporal resolution (*fs* = 140 Hz) from a large imaging volume  ~ 10 body lengths per linear dimension, and include both square and cylindrical arenas. This dataset offers a critical resource for biologists seeking to understand the neural basis of social behavior, for machine learning researchers working to improve posture tracking, and for the broader quantitative understanding of natural behavior.

## Introduction

Zebrafish (*Danio rerio*) has emerged as a valuable model organism for studying social behavior due to its genetic tractability, relatively simple nervous system, and complex social interactions that include aggressive behaviors, particularly among males^1–6^. While traditional behavioral studies have provided qualitative descriptions of zebrafish aggression, there remains a critical need for high-resolution, quantitative datasets that capture the fine-grained dynamics of these interactions. Previous tracking methods have been largely limited to single-point tracking^7^ or two-dimensional analysis^8,9^, which fail to capture the rich biomechanical and postural information essential for understanding the physical principles underlying social behaviors^10^.

Here we present an extensive dataset of zebrafish aggressive interactions in health and disease, tracked for long durations at high spatial and temporal resolution. We imaged the behavior of pairs of zebrafish in a total of 173 experiments, in wild-type (WT) animals as well as two mutants that manifest behavioral disorders: Rett syndrome (RTT) and aggression-boldness syndrome. Our methodology employs three synchronized high-speed cameras capturing behavior at 140 frames per second (FPS) for 5 hours, allowing detailed reconstruction of three-dimensional postures for interacting fish pairs across behavioral epochs. By simultaneously tracking multiple anatomical landmarks (head, pectoral region, and tail) while maintaining individual identity throughout extended recordings, we provide a dataset that enables fine-grained analysis of the physical dynamics governing social encounters.

The dataset presented here supports a range of research applications: (1) a baseline for normal aggressive behavior in WT zebrafish against which disease models can be compared, particularly for neurodegenerative conditions where movement abnormalities may be subtle; (2) testing theories of social dominance; (3) a methodological template for high-precision behavioral tracking that can be adapted to other model organisms and experimental paradigms; and (4) a substrate on which to implement and test improvements in tracking workflows (e.g., for imputation of missing data^11^). With our contributed empirical data we aim to advance both the quantitative understanding of social interactions^12^, and that of behavior more generally^13,14^.

## Methods

### Zebrafish husbandry and experiments

Three generations of Okinawa wild-type (Oki WT) zebrafish were used (450 fish in total) over the span of 18 months, with a ratio of 1:2 male to female. The fish were housed in 3 L tanks with 6 males and 12 females in each tank. The fish age varied from 6 to 14 months. Fish husbandry and maintenance were conducted according to general methods for zebrafish care and experiments^15^. The fish were kept on a 14 h light/10 h dark cycle with light on at 9:00 a.m. Each individual participated up to four times in a recording. Experiments were conducted as follows: 24–72 hours before recording, two males (or females) of similar size were collected from two different group tanks and separately isolated in individual 1.8 L tanks. This period of isolation was motivated by previous research showing that zebrafish memory fades after 24 h^16^, which negates the effect of any existing hierarchies that may have existed in the housing tanks. Immediately before each recording, freshly prepared system water (pH 7.0, 300 *μ*S/cm) was cooled to 28.6 ^°^C to be as close as possible to the housing conditions in the fish maintenance tanks. The water was filtered through a net to keep debris out of the experimental tank. Once the experimental tank was filled with water, an interior cage was placed, and air bubbles were removed. The fish were then moved to the experimental tank using nets. Most of the recordings started between 9-10 a.m., or occasionally in the afternoon. Experiments were performed on fasting fish, skipping the scheduled morning feeding. After each recording, fish were moved to a new group housing tank with the same male-to-female ratio as a way of tracking the number of times each individual participated in a recording. Besides WT, our experiments include two mutant types. A zebrafish model of RTT that exhibits motor and behavioral abnormalities, carrying the *mecp2* mutant allele (sa21196, ZFIN ID ZDB-ALT-131217-5751)^17^, and a fgf receptor 1a (*fgfr1a*) mutant allele (spiegeldanio, t2227 (t3R705H), ZFIN ID ZDB-ALT-100108-1) exhibiting elevated aggression^18^. A low number of mutants prevented them from being collected from different housing tanks (as was the case with the WT), but the isolation protocol for each individual was the same.

### Ethics statement

Zebrafish experiments were conducted in accordance with the OIST Animal Care and Use Program, adhering to the principles outlined in the Guide for the Care and Use of Laboratory Animals by the National Research Council of the National Academies. Our experimental procedures were approved by the Association for Assessment and Accreditation of Laboratory Animal Care (AAALAC). All experimental protocols received authorization from the OIST Institutional Animal Care and Use Committee (Protocols: ACUP-2022-005-2, ACUP-2023-018, ACUP-2023-022-2, ACUP-2025-032). This study was conducted and reported in accordance with the ARRIVE Essential 10 guidelines.

### Experimental set-up

**Fig. 1:**
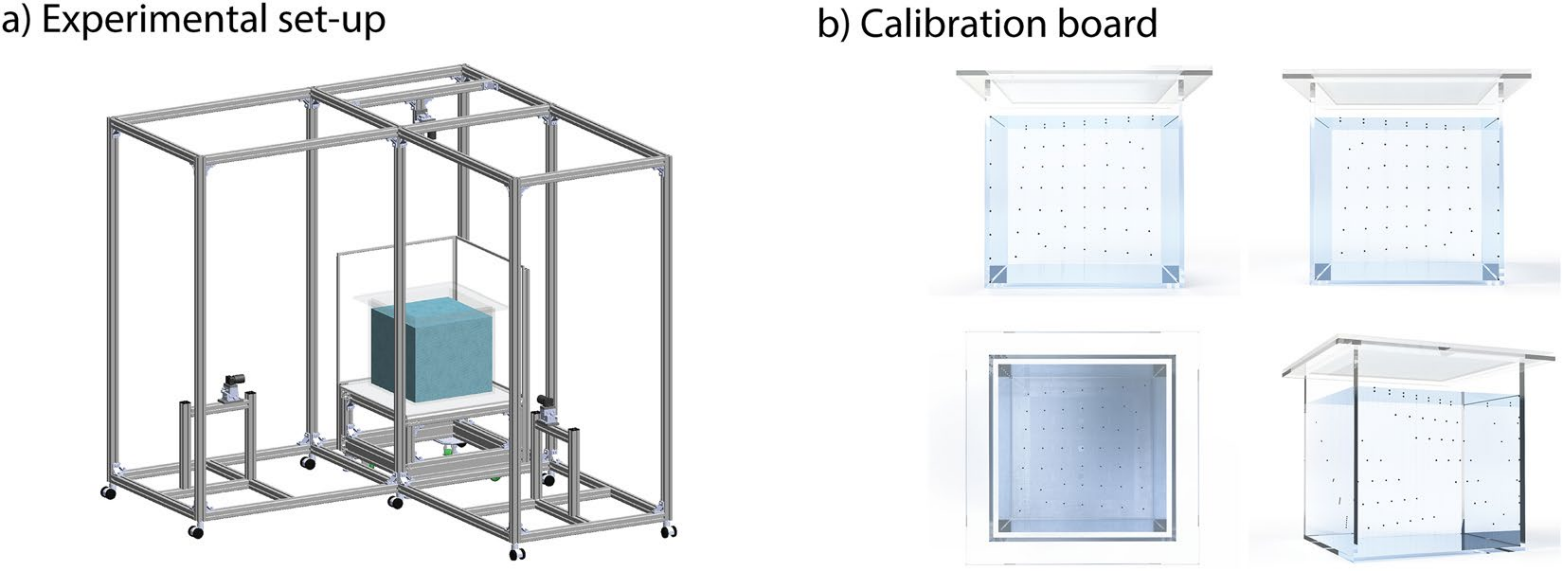
**a**) Illustration of the imaging system, composed of mounting gear, an observation tank, and three cameras. We positioned one camera on top and two on the sides. The use of two side cameras helps in resolving occlusions, especially during the close interactions that are common in social behavior. (**b**) We show three orthographic views and a rendered perspective of the calibration bead structure in the observation tank. We use images from the three orthogonal views to construct calibration models. The calibration board consisted of a total of 49 beads, which were attached to the board’s top with a thread made from fluorocarbon fish wire.

We leverage an imaging system described previously^12^ and incorporate improvements to increase the spatiotemporal resolution and throughput. We recorded zebrafish behavior using three synchronized machine-vision cameras positioned along orthogonal views of a 40 × 40 × 44 cm tank (Fig. 1a). The cameras (CM3-U3-13Y3M-CS, Teledyne Vision Solutions) contained Onsemi PYTHON 1300 sensors with 1280 × 1024 pixels and 12 mm focal length lenses (12VM412ASIR, TAMRON). Each camera view utilized an LED square panel for backlight illumination, creating distinct visual contrast between the background and zebrafish, while a flicker-free LED driver maintained uniform frame intensity. White matte-type diffuser sheets on the bottom and two outer sides of the tank enhanced lighting uniformity, with light intensity measuring on average 2300 lx. To eliminate reflection artifacts, an interior cage composed of an acrylic frame and fluorinated ethylene propylene film (127 *μm* thickness) was used. The film exhibits optical transparency in water with a refractive index (*n* ~ 1.34) nearly identical to water (*n* ~ 1.33) and high transparency (>90%). Two cage shapes were used: cubic and cylindrical. The measurements of the cubic cage were 25 × 25 × 25 cm, while the cylindrical cage had a diameter of 25 cm and a height of 25 cm. Given that adult zebrafish are approximately 2.5 cm in length, the imaging volume offered roughly 10 body lengths per spatial dimension, allowing for comprehensive tracking of movement and social interactions in three-dimensional space. The cameras recorded at 140 FPS in 8-bit grayscale format, with data stored using FFmpeg (codec nvenc H.264), compressed through an NVIDIA GPU (Quadro RTX A5000, NVIDIA). An Arduino board (Arduino MEGA2560) controlled frame-wise camera synchronization through external triggers. A Python package, campy (https://github.com/ksseverson57/campy), managed all recording operations. White cardboard shielding covered the entire recording setup to eliminate external visual disturbances from the surrounding environment.

### Calibration

To map the camera to the real-world coordinates, we calibrated the imaging volume using a custom calibration board composed of 49 beads with a bead diameter of 2 mm (Fig. 1b). We submerged the calibration beads structure in the tank and photographed it in four different orientations. We then automatically identified the centroid of each bead in all captured images. Using these 2D image coordinates and the known 3D real-world coordinates of the beads, we regressed five functions: (1) Two 3D-mapping functions: One function maps the set of 2D image coordinates from three orthogonal views (XZ, YZ, XY) to the corresponding 3D real-world coordinates (XYZ). The second function performs the opposite mapping (XYZ -> [XZ, YZ, XY]). (2) Three view-mapping functions: These functions map 2D coordinates from any two orthogonal views to the corresponding 2D coordinate in the third view (e.g., mapping coordinates from the XZ and YZ views to the XY view). The two 3D-mapping functions were modeled using a second-order polynomial regression. The three view-mapping functions were modeled using third-order polynomial regression. Calibration was conducted weekly and after any incidental movement of the cameras (e.g., after an earthquake).

### 3D posture tracking

**Fig. 2:**
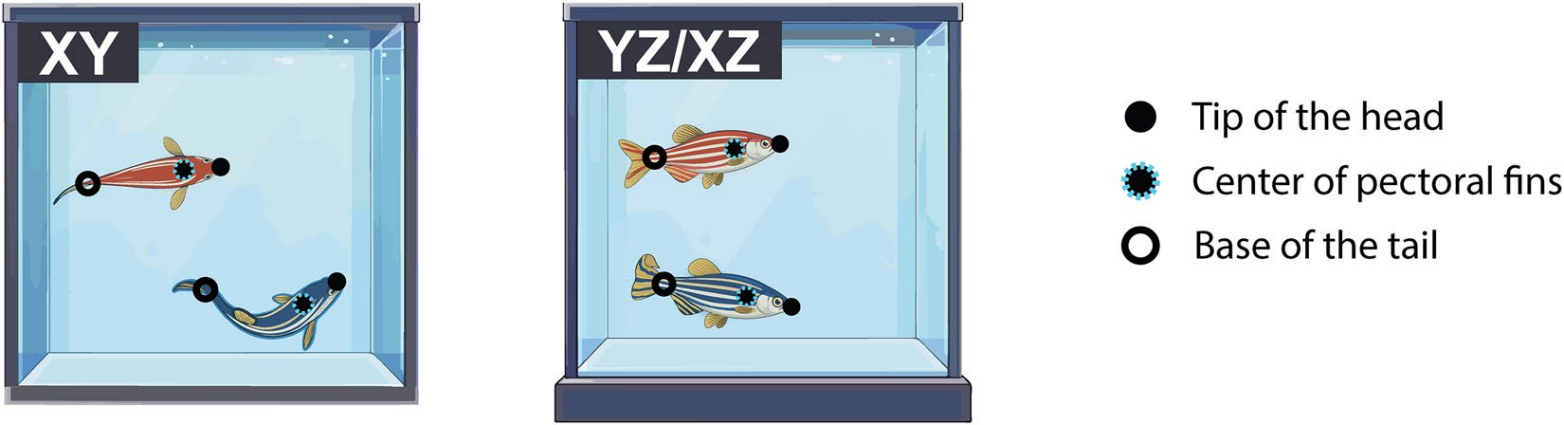
Illustration of the three body points tracked in our experiments: the tip of the head, the center of the pectoral fins, and the base of the tail. XY: top camera; YZ/XZ: side cameras.

We tracked the 3D posture of each fish by detecting the three following body points: the tip of the head, the center of the pectoral fins, and the base of the tail (Fig. 2). Together, these three body points define the skeleton of a fish in our experiments.

**Fig. 3:**
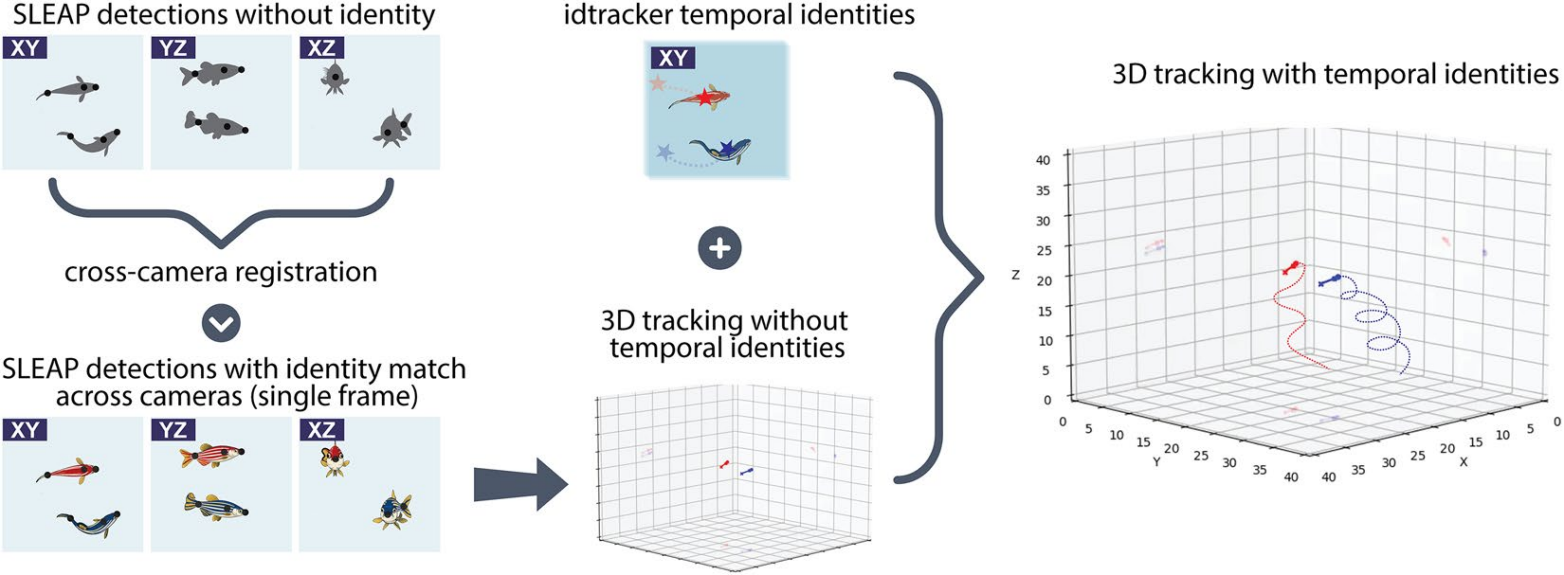
Overview of our tracking workflow. We used two complementary machine vision tools, SLEAP and idtracker, to produce multi-body-point tracking across identified individuals in 3D. We applied SLEAP to detect the 2D body points in each of the three different camera views, and we used idtracker for consistent fish identities across the whole experiment. We show SLEAP detections of the three different body points as black circles overlaid on the fish illustrations. We visualize the centroid of the fish provided by idtracker as a filled star overlaid on the individual fish.

We used two complementary tools that allowed us to track the 3D posture of each fish throughout the whole video while maintaining the correct identities: SLEAP^19^ and idtracker.ai^20^ (hereinafter referred to as idtracker) (Fig. 3). SLEAP is an open-source, deep-learning-based framework for multi-animal posture tracking, while idtracker is an open-source deep-learning-based software for tracking the identities of multiple individuals in a video (without posture information). SLEAP follows a supervised learning approach, where frames need to be labeled and used as training examples, while idtracker detects “blobs” and tracks their identities through the unsupervised characterization of morphological features. For SLEAP, we manually annotated 1,200-1,500 frames per camera view, which were then used to train three individual (camera-specific) inference models that were used in all the subsequent experiments. An overview of our tracking workflow is illustrated in Fig. 3.

After recording an experiment, we ran SLEAP on all three camera views (XY, YZ, XZ), resulting in a maximum of two individual 2D skeletons for each camera, for each frame. As SLEAP detections are conducted in 2D, independently for each camera view, it was not immediately apparent how fish identities match across the three different cameras (for a single frame). Therefore, to correctly assign the corresponding fish identities in all three camera views, we used an exhaustive search method that uses two source cameras as starting points and calculates the cost of reprojecting the fish skeletons back to these source cameras, after projection to the third camera through the calibration models. The exhaustive search was conducted as follows: for each possible fish identity permutation, we calculated the reprojection cost for one of the source cameras as the average cost of reprojecting the two fish skeletons from two camera views to the third one and back to the selected source camera. The cost is the average distance, in pixels, of the reprojected body point locations to the body point locations in the source camera view. Costs were also averaged over the fish identities and the two source cameras, resulting in a single cross-camera registration cost for each permutation.

**Fig. 4:**
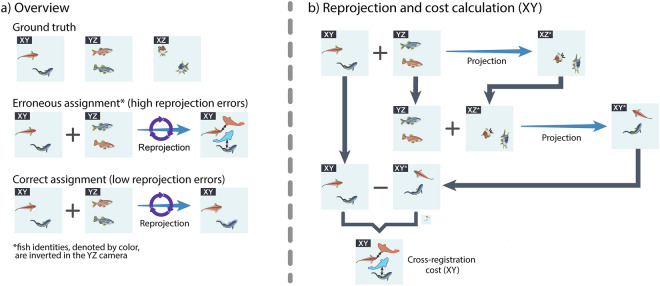
Illustration of the cross-camera registration methodology used for assigning fish identities across all three camera views. The approach used in this work is an exhaustive search method that tests all four different combinations of fish identities for two source cameras. For each combination of fish identities in the two source cameras, the reprojection errors are calculated as the distance (in pixels) of the reprojected skeletons to the original skeletons in each of the source cameras. In the examples shown here, fish identities are denoted with a different fish color (blue or orange). (**a**) Overview of our methodology. If the fish identities are appropriate, reprojection of skeletons through the calibration models should result in skeletons that are close to the skeletons in the source camera views (shown here for XY camera). In the case of “erroneous assignment”, the fish identities in the YZ camera have been inverted, while the identities in the “correct assignment” case are equal to the ground truth. (**b**) An example of how cross-camera registration cost is calculated through the reprojection of skeletons, for a single source camera (XY). Shown here is the case of erroneous assignment in a). Since this is an erroneous assignment, the resulting projections through the calibration model result in incorrect and/or distorted fish skeletons (indicated as XZ* and XY*).

**Fig. 5:**
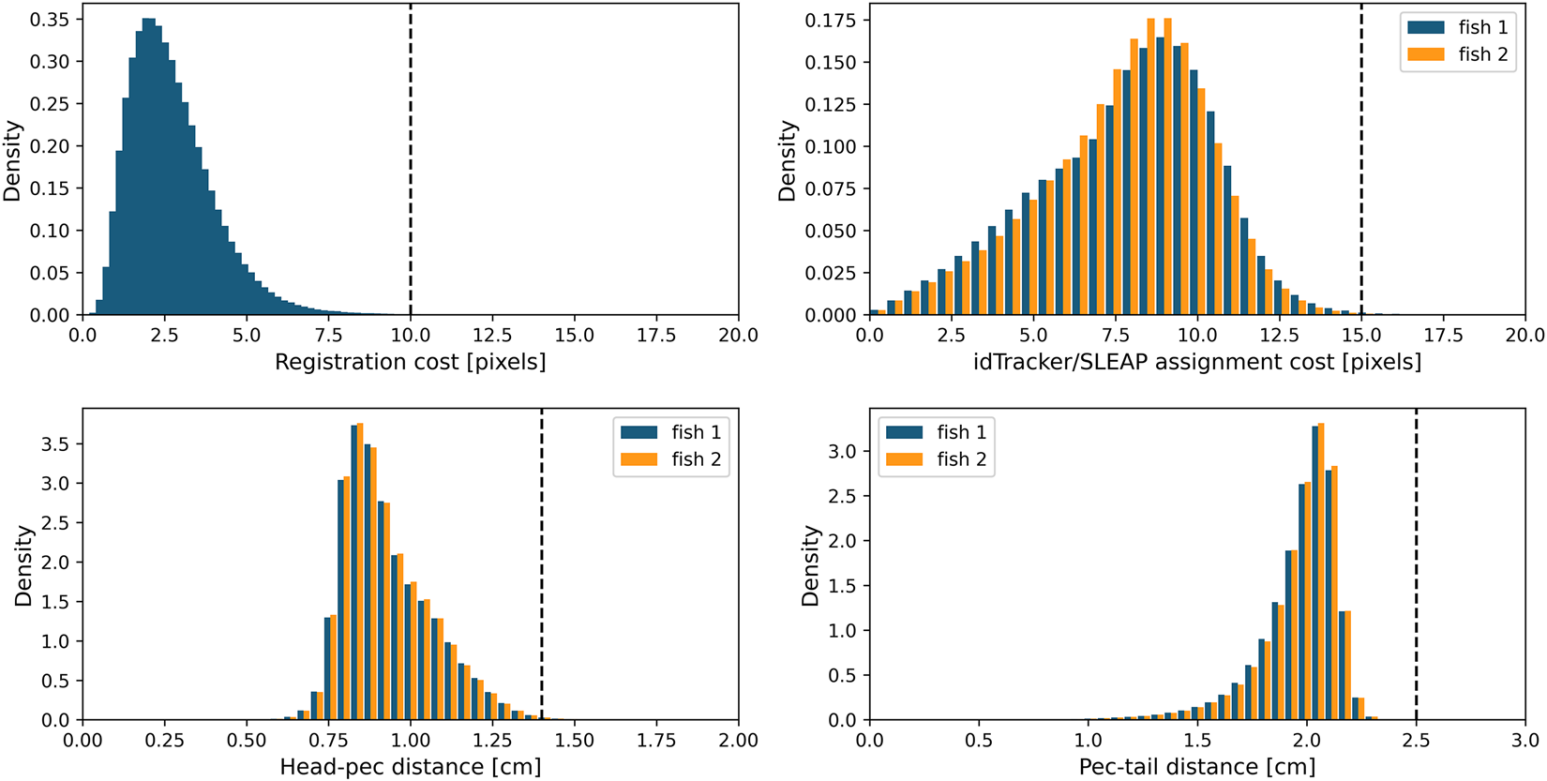
Illustration of thresholds used to minimize spurious skeleton detections (data from a representative experiment). **Top left:** Distribution and threshold used during the cross-camera registration. Cross-camera registration finds the best permutation for matching the two skeletons in all three cameras, for a single frame. **Top right:** idtracker/SLEAP registration costs and thresholds. The pec point of the SLEAP skeleton was matched against the centroid detection from idtracker in order to assign fish identities across the whole video. **Lower left:** Head-pec distance distribution and thresholds for fish skeletons. **Lower right:** Pec-tail distance distribution and thresholds for fish skeletons. All thresholds used are shown with the dashed vertical line. Note, fish body length is approximately 90 pixels.

Our cross-camera registration method takes advantage of the fact that, if the assignment of the fish identities is correct, reprojecting the skeletons back to the original camera view should result in the reprojected skeletons being close to the original skeletons. Alternatively, if the fish identities are wrong, reprojection would result in fish skeletons that are far away from the original skeletons (Fig. 4). In our experiments, the permutation with the lowest cross-camera registration cost was defined as the correct pairing of the fish identities. We found that the cross-camera registration cost was centered at around 3 pixels (Fig. 5). To maintain high-quality posture tracking, skeletons with cross-camera registration costs greater than 10 pixels were set as N/A. Once the fish identities were matched, we combined the body point locations of all cameras and derived the 3D coordinates of each fish through the calibration model.

To determine fish identity throughout the whole video, we first ran idtracker on the XY camera view. We then merged the SLEAP-based 3D posture of each fish with the idtracker identities by assigning the 3D skeleton of a fish to the idtracker identity whose centroid location was closest to the pec point in the skeleton found by SLEAP. As the centroid detected by idtracker does not necessarily match exactly with the pec point of the SLEAP skeleton, some variability in the corresponding points is expected. Skeletons where the distance between the centroid and the pec point was larger than 15 pixels were set as N/A. More information on the SLEAP/idtracker point distances is provided in Section Technical Validation.

### Postprocessing

We accounted for and removed data in which any of the following cases occurred: identity swap, single-fish jump, single-bodypoint jump. Identity swap is defined as a simultaneous jump in the velocity of both fish. Single-fish jump is defined as the jump of all three body points of a single fish (when, for example, the other fish was undetected). Single-body-point jump is defined as the sudden jump in velocity of a specific body point. Such single-point jumps frequently occurred when fish were close together, due to the misidentification of body parts through the SLEAP models. All of these erroneous cases were set as N/A. In addition, to minimize data loss from the post-processing, we closed gaps up to seven frames with linear interpolation followed by a second-order Savitzky-Golay filter with a window of nine frames.

## Data Records

The dataset is available in Zenodo^21^ (10.5281/zenodo.17190142). The dataset comprises three archive files containing the tracks of the two fish in CSV format for 173 experiments, each lasting 5 hours. A total of 152 experiments are WT, 7 of which are female-female pairs, and 145 experiments are male-male pairs. In addition, 9 experiments were conducted with *mecp2* mutant pairs, and 12 with *fgfr1a* mutant pairs. The tracks of each fish are separated in the coordinates of the fish head, pec, and tail. The files that can be found in the repository are as follows:

- tracks_wt.zip: This file contains the tracks of the WT experiments.
- tracks_mecp2.zip: This file contains the tracks of the mecp2 experiments.
- tracks_fgfr1a.zip: This file contains the tracks of the fgfr1a experiments.
- source_videos_Zebrafish20250204_0944.zip: This file contains the source videos for one experiment.
- sample_video_Zebrafish20250204_0944.mp4: This is a sample video (1 minute) containing all three camera views overlaid with the 2D SLEAP predictions, as well as the final 3D locations of the fish.
- metadata.csv: This is a CSV file containing the metadata of the experiments. The columns included are the experiment ID, the date of the experiment, the sex of the fish, the cage type (circular or square), and the fish genotype.

## Technical Validation

### Quality assurance

**Fig. 6:**
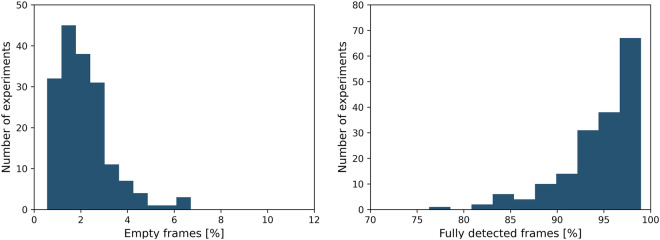
Tracking quality summary statistics. Empty frames are defined as frames where there is no detected bodypoint for either fish, while fully detected frames correspond to frames with full 3D information for both fish (i.e., all three body points).

Figure 5 illustrates the distributions of the cross-camera registration costs, SLEAP/idtracker assignment costs, head-pec distance, and pec-tail distance of the identified skeletons for a representative tracking experiment. The cross-camera registration costs were well below 10 pixels, which was used as a threshold to remove potentially spurious assignments. Matching SLEAP to idtracker detections was another source of potentially spurious identity assignments, and this was accounted for by removing detections where the SLEAP/idtracker assignment cost was larger than 15 pixels. Here, some variability is expected, as SLEAP detects the three defined body points, while idtracker tracks the centroid of the fish. Lastly, abnormally long head-pec and pec-tail distances were classified as erroneous detections and removed from the data (head-pec threshold: 1.4 cm, pec-tail threshold: 2.5 cm). Note, removing spurious detections did not necessarily result in a reduction of the tracked frames, due to the postprocessing that included interpolation up to seven frames. Overall, our tracking procedure resulted in high-quality 3D posture tracks of both fish engaged in social interactions. Figure 6 shows the percentage of frames with full information (all three body points for both fish) or no information (empty frames).

### Interpretable behavioral analysis

**Fig. 7:**
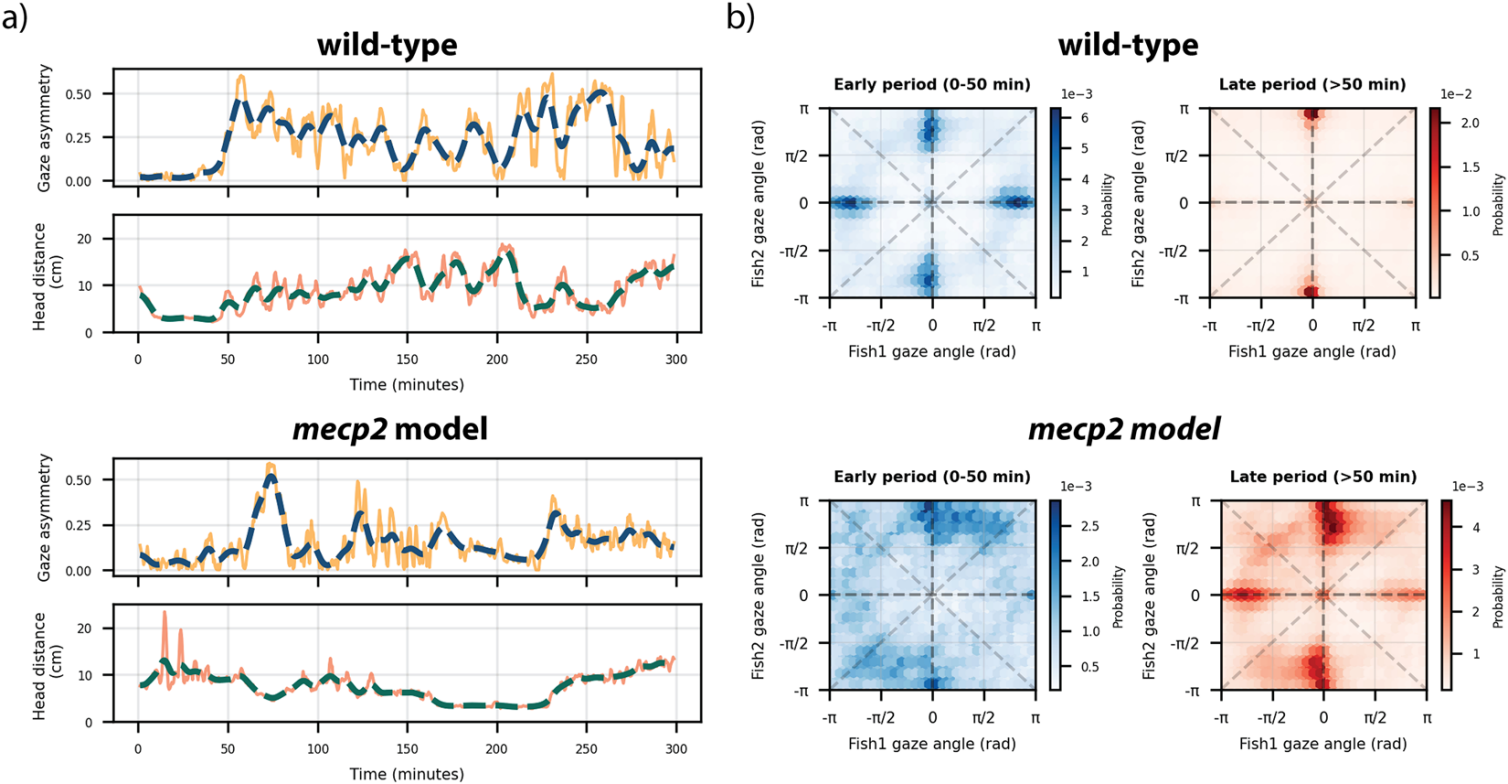
Example of gaze asymmetries for wild-type and *mecp2* mutant fish. (**a**) Temporal profile of gaze asymmetry and head-to-head distance. Smoothed data is presented with thick, dashed blue and green lines. (**b**) 2D histograms of the gaze angles for early (0–50 min) and later (>50 min) phases of the experiments. In the initial stages of the example wild-type experiment, both fish aggressively interact with each other, resulting in a symmetric profile of the gaze angles. In the later phases of the experiment, after dominance is established only the winner fish consistently looks at the loser^12^, resulting in an asymmetric profile of the gaze angles. For the *mecp2* mutants, the gaze angles remain much more symmetric across the organisms.

To demonstrate that our experimental process and tracking approach results in high-quality data that can uncover fine-grained interactions, we conducted a preliminary analysis of gaze asymmetries in two experiments, one with WT fish and one with *mecp2* mutant pairs. This analysis is motivated by recent work which demonstrated a sudden asymmetry in the relative orientations of the two fish, reflecting the establishment of dominance^12^. Here, we define gaze asymmetry as:

1 $${A}_{{\rm{gaze}}}=\frac{| {N}_{1\to 2}-{N}_{2\to 1}| }{{N}_{{\rm{total}}}}$$

where *N*_1→2_ and *N*_2→1_ denote the number of frames in which fish1 and fish2, respectively, are oriented toward the other fish within a  ± 20 degrees threshold, and *N*_total_ is the total number of frames in a 2-minute analysis window. In Fig. 7a we show that in the initial phase (first 50 minutes) of a WT experiment, gaze asymmetry is low, as the fish are engaged in close-contact, mutually aggressive interactions. As the recording progresses, asymmetry rises due to the establishment of dominance. On the contrary, the *mecp2* mutants do not exhibit such a consistent asymmetry in gaze angles.

In Fig. 7b we show the two-dimensional probability of the gaze angles. This representation captures the distribution of mutual orientation patterns across the observation window. In the later period of the WT experiment (>50 min), only one fish is consistently oriented towards the other, reflecting the establishment of dominance. On the other hand, for our *mecp2* experiment, both fish are clearly oriented towards each other even at the later stages of the experiment.

## Usage Notes

The current dataset comprises 173 experiments involving male-male and female-female zebrafish pairs engaged in competitive interactions, with both WT and mutant pairs. Our dataset differs from previous studies in: (1) tracking 3D posture, (2) high frame rate of 140 FPS, (3) long (5-hour) duration of the recordings covering multiple behavior epochs, and (4) extensive number of recordings. This data extends previous works^3,22^ and enables the investigation of the fine mechanisms of competitive social behavior by integrating spatial and postural information (e.g.^23^).

Zebrafish are increasingly being used as models for human neurodegenerative diseases, such as Parkinson’s^24^ and Alzheimer’s disease^25^, due to their low cost and high degree of genetic similarity with humans^2^. For comparative analysis, in addition to healthy, WT fish, we have also imaged the behavior of two genetically-modified zebrafish models, a model for RTT (*mecp2* mutant) and a model exhibiting increased aggression (*fgfr1a* mutant).

The reader should note that not all experiments include a fight, and not all fights conclude with the establishment of dominance. Fish may also engage in multiple fights during the course of the experiments. In addition, in some experiments, dominance is apparent from the start of the experiment, in agreement with our previous observations^12^. Users should also note that despite our comprehensive quality control pipeline, some residual identity swaps or body-part misidentifications may remain in the data, particularly during close-proximity aggressive encounters.

This work builds upon our “physics of behavior” approach (see e.g.^26–28^), which combines novel quantitative measurement with principles from statistical physics and dynamical systems theory to understand animal behavior as emergent phenomena governed by underlying physical laws. This perspective has previously revealed interpretable behavioral patterns in organisms ranging from *C. elegans*^29^ to bees^30^. By extending this approach to zebrafish social interactions, we aim to bridge the gap between biomechanics, neuroscience, and physical theory, while providing a medium for machine learning researchers to implement and test their algorithms on (behavior analysis, winner prediction, etc.).

## Data Availability

All tracked experiments, as well as the corresponding metadata and sample video files, are available in Zenodo^21^ (10.5281/zenodo.17190142). Further details are provided in the Data Records section.

## Code availability

Code is available on GitHub at the following address: https://github.com/deligkarisk/Zebrafish_3D_Tracking_Workflow.
